# MAMP (microbe-associated molecular pattern) triggered immunity in plants

**DOI:** 10.3389/fpls.2013.00139

**Published:** 2013-05-16

**Authors:** Mari-Anne Newman, Thomas Sundelin, Jon T. Nielsen, Gitte Erbs

**Affiliations:** Department of Plant and Environmental Sciences, University of CopenhagenFrederiksberg, Denmark

**Keywords:** innate immunity, MAMPs, Flg22, EF-Tu, Ax21, PGN, LPS, Chitin

## Abstract

Plants are sessile organisms that are under constant attack from microbes. They rely on both preformed defenses, and their innate immune system to ward of the microbial pathogens. Preformed defences include for example the cell wall and cuticle, which act as physical barriers to microbial colonization. The plant immune system is composed of surveillance systems that perceive several general microbe elicitors, which allow plants to switch from growth and development into a defense mode, rejecting most potentially harmful microbes. The elicitors are essential structures for pathogen survival and are conserved among pathogens. The conserved microbe-specific molecules, referred to as microbe- or pathogen-associated molecular patterns (MAMPs or PAMPs), are recognized by the plant innate immune systems pattern recognition receptors (PRRs). General elicitors like flagellin (Flg), elongation factor Tu (EF-Tu), peptidoglycan (PGN), lipopolysaccharides (LPS), Ax21 (Activator of XA21-mediated immunity in rice), fungal chitin, and β-glucans from oomycetes are recognized by plant surface localized PRRs. Several of the MAMPs and their corresponding PRRs have, in recent years, been identified. This review focuses on the current knowledge regarding important MAMPs from bacteria, fungi, and oomycetes, their structure, the plant PRRs that recognizes them, and how they induce MAMP-triggered immunity (MTI) in plants.

## Introduction

Bacteria, fungi, oomycetes, and viruses attack plants in an attempt to gain nutrients from them. During the course of evolution both plants and pathogens have evolved features to combat each other; the plant is equipped with sophisticated and rapidly mounted defense mechanisms, while their cognate pathogens have developed counterstrategies to overcome those defenses, the so called “arms race” between plant and pathogens (Bent and MacKey, 2007). The interplay between the plant defense systems and its suppression by pathogens has been portrayed as a “zigzag model” by Jones and Dangl (2006). This model proposes that the plants' immune responses consist of two branches. The first line of defense in plants is the recognition of conserved molecules characteristic of many microbes. These elicitors are also known as microbe- or pathogen-associated molecular patterns (MAMPs or PAMPs) (Table 1). MAMPs are essential structures for the microbes and are for that reason conserved both among pathogens, non-pathogenic and saprophytic microorganisms. MAMPs are recognized by pattern recognition receptors (PRRs), which are localized on the surface of plant cells; this first phase of defense induction is called MAMP-triggered immunity (MTI) (Ausubel, 2005; Jones and Dangl, 2006). All known plant PRRs are plasma membrane-localized receptor-like kinases (RLKs) or receptor-like proteins (RLPs) with modular functional domains. RLKs contain an extracellular domain (ECD), a single-pass transmembrane (TM) domain, and an intracellular kinase domain. RLPs contain an ECD and a TM but have only a short cytosolic domain without an obvious signaling domain (Table 1). Notably, in contrast to mammals, no intracellular nucleotide-binding-leucine-rich repeat (NB-LRR) protein recognizing a MAMP has yet been identified in plants (Maekawa et al., 2011). Bacterial effector proteins, injected directly into the host plants' cytoplasm via the pathogens type III secretion system (TTSS), have been demonstrated to suppress MTI (Jamir et al., 2004; He et al., 2006; Nomura et al., 2006), resulting in effector-triggered susceptibility (ETS). The second line of the plants' defense is direct or indirect recognition of a given effector through a set of plant resistance (R) gene products resulting in effector-triggered immunity (ETI) (Jones and Dangl, 2006); also named the gene-for-gene interaction as early as 1942 by Flor. ETI is generally an accelerated and amplified MTI response, and as such it is an effective defense response (resistance) that in most cases leads to a localized cell death, known as the hypersensitive response (HR). The majority of the R proteins are intracellular receptor proteins of the NB-LRR type. In most cases the interaction between NB-LRRs and the effectors are indirect (van der Biezen and Jones, 1998).

**Table 1:** **Microbe-associated molecular patterns (MAMPs) and Damage-associated molecular patterns (DAMPs)**.

**Name**	**Corresponding plant receptor (PRR)**	**References**
**MAMPs**		
Flagellin (Flg; flg22)	FLS2 (*Arabidopsis*)	Felix et al., 1999; Gómez-Gómez et al., 2001
Elongation factor TU (EF-Tu; elf18/26)	EFR (*Arabidopsis*; Brassicaceae)	Kunze et al., 2004
Peptidoglycan (PGN)	Lym1 and Lym3 (*Arabidopsis*)	Gust et al., 2007; Erbs et al., 2008a; Willmann et al., 2011
Lipopolysaccharide (LPS)	Not identified	Newman et al., 1995
Bacterial cold shock proteins (RNP1 motif)	Not identified	Felix and Boller, 2003b
Bacterial superoxide dismutase (Sod)	Not identified	Watt et al., 2006
Activator of XA21 (Ax21)	XA21 and XA21D (rice)	Song et al., 1995; Wang et al., 1998; Lee et al., 2009
Beta-Glycan (GE)	GEBP (putative receptor soyabean)	Darvill and Albersheim, 1984; Umemoto et al., 1997
Chitin	CeBip and CERK1 (rice); AtCERK1 (*Arabidopsis*)	Felix et al., 1993; Kaku et al., 2006; Miya et al., 2007;Shimizu et al., 2010
Avirulence on Ve1 tomato (Ave1)	Ve1 (putative tomato receptor)	Kawchuk et al., 2001; Thomma et al., 2011;de Jonge et al., 2012
Xylanase (EIX)	EIX (tomato)	Bailey et al., 1990; Ron and Avni, 2004
Pep-13 (An oligopeptide of 13 amino acids from *P*. *mega-sperma*)	Not identified	Nürnberger et al., 1994
Cellulose-binding elicitor lectin (CBEL) from *Phytophthora*	Not identified	Mateos et al., 1997; Séjalon-Delmas et al., 1997 Gaulin et al., 2006
**DAMPs**		
Systemin	Not identified	Narváez-Vásquez and Ryan, 2004
Pep1 (23 aa part of a cytosolic protein from *Arabidopsis*)	PEPR1 (*Arabidopsis*)	Huffaker et al., 2006; Yamaguchi et al., 2006
Oligogalacturonides (OGs)	WAK1 (*Arabidopsis*)	Nothnagel et al., 1983; Brutus et al., 2010
Cutin	Not identified	Schweizer et al., 1996; Kauss et al., 1999

MAMP-induced defense responses include the production of reactive oxygen species (ROS, also called the oxidative burst), production of reactive nitrogen species such as nitric oxide (NO), alterations in the plant cell wall, induction of antimicrobial compounds and the synthesis of pathogenesis-related (PR) proteins. ROS and NO can act in signaling and have direct antimicrobial effects. ROS can also drive oxidative cross-linking of polymers in the plant cell wall to strengthen it against degradation, which may restrict pathogen spread. Other alterations in the plant wall include the deposition of the β-(1–3) linked glucan callose. PR proteins comprise a number of families that include enzymes, such as β-(1–3) glucanase and chitinase, which can directly attack pathogen structures, antimicrobial peptides and small proteins, and PR1, which is of unknown function [for reviews, see Hammond-Kosack and Jones (1996); Greenberg (1997); Lamb and Dixon (1997); Nürnberger and Kemmerling (2006)]. The induction of MTI in plants has been most extensively studied using the small peptides flg22 and elf18 derived from the bacterial MAMPs flagellin (Flg) and the translation elongation factor Tu (EF-Tu), respectively (Felix and Boller, 2003a; Zipfel et al., 2006). Bacterial glycoconjugates, such as the peptidoglycan (PGN), which provides rigidity and structure to the cell envelopes of both Gram-negative and Gram-positive bacteria (Erbs et al., 2008a; Willmann et al., 2011), and lipopolysaccharides (LPS) from the outer membrane of Gram-negative bacteria have been found to act as elicitors of plant innate immunity (Silipo et al., 2005; Erbs and Newman, 2012). Oligosaccharides derived from cell wall polymers of fungi and oomycetes also act as MAMPs. Fungal chitin and its degradation products N-acetyl-chito-oligosaccharides, i.e., chitin oligomers induce various defense responses in both monocot and dicot plants (Kaku et al., 2006; Miya et al., 2007). In the oomycetes, the cell walls are composed of β-glucans and cellulose, rather than chitin, as in the fungi. Some of the earliest work on the role of glycosylated compounds in triggering plant defenses has examined the effects of β-(1→3/1→6)-linked glucans from the cell walls of *Phytophthora megasperma* f. sp. *glycinea* on the induction of phytoalexin accumulation in soybean [reviewed in Cheong and Hahn (1991)]. In the plant-virus interactions no conserved viral MAMP has been identified so far, and the primary plant defense is thought to be based mainly on RNA silencing (RNAi). By analogy with the zigzag model, viral-derived double-stranded RNA (dsRNA) is regarded as the MAMP inducing RNAi, a general plant defense mechanism or the MTI. To counteract this defense, plant viruses express RNA silencing suppressors (RSSs), many of which bind to dsRNA and attenuate RNAi (Csorba et al., 2009; Ruiz-Ferrer and Voinnet, 2009).

This review will focus on some of the important MAMPs from bacteria, fungi, and oomycetes and review the current knowledge of their structure, how they are recognized and how they induce MTI in plants. We include the slightly more unusual MAMP Ax21 from the rice pathogenic bacteria *Xanthomonas oryzae* pv. *oryzae* (*Xoo*). MAMPs in general activate MTI directly via their respective plant receptors, whereas Ax21 is secreted out of the bacterium via the type-I secretion system (TOSS), where it is then recognized by the rice receptor XA21, and induction of MTI follows. Finally, we will briefly describe damage-associated molecular patterns (DAMPs) of plant origin, which also induce MTI in the plant.

## Bacterial MAMPs

### Flagellin (Flg)

Flagella are essential structures for the pathogenic bacteria as they provide motility and often increase adhesion of the bacteria to its host. Flg, the main building block of bacterial flagella, is well-established as a major activator of innate immunity in animals [reviewed by Ramos et al. (2004)]. Some of the first MAMP recognition studies in plants were carried using Flg. Studies in mammals have shown that at least one of the conserved domains in the N-terminal and C-terminal part of the bacterial Flg, found to be involved in bacterial motility as well, is recognized by Toll-like receptor 5 (TLR5) (Hayashi et al., 2001; Smith et al., 2003). Studies in *Arabidopsis*, tomato, and other plants, revealed that plants respond to a highly conserved domain in the N-terminal part of the bacterial Flg, a 22 amino acid (aa) peptide, flg22 (Felix et al., 1999). In order to identify the gene involved in recognition and transduction of the flg22 elicitor signal, Gomez-Gomez and Boller (2000), used a genetic approach to screen *Arabidopsis* mutants after flg22 treatment and isolated several Flg sensing 2 (FLS2) mutants, which mapped to the FLS2 locus on chromosome 5. FLS2 belongs to the RLK family and has an ECD with 28 LRRs, a TM domain, and an intracellular serine/threonine kinase domain. No high-affinity binding site was found, after treatment with a radiolabeled derivative of flg22, in the Flg insensitive *Arabidopsis* ecotype Ws-0 and in plants carrying mutations in the LRR domain of the FLS2 gene, indicating a role for LRR in Flg binding (Gomez-Gomez and Boller, 2000; Bauer et al., 2001). Later work revealed that both an extracellular LRR domain and kinase activity of FLS2 were necessary for high affinity binding and binding specificity for Flg (Gómez-Gómez et al., 2001). Chinchilla et al. (2006) showed the specific interaction of flg22 with FLS2 in *Arabidopsis*. The recognized domain within Flg is not the same for all plant species. For example, flg15, an N-terminally shortened version of flg22, was shown to be highly active in tomato, while it only elicits immune responses at higher concentrations in *Arabidopsis*. Rice is able to recognize flg22, but its defense response is greater to the full length Flg (Takai et al., 2008). The functionality of the FLS2 receptor was tested by heterologous expression of the *Arabidopsis* FLS2 receptor in tomato cells. In these expression studies, tomato cells gained the Flg perception system characteristic for *Arabidopsis*, demonstrating that FLS2 represents the PRR that determines the specificity of Flg perception (Meindl et al., 2000; Bauer et al., 2001; Chinchilla et al., 2006). The difference in recognition of the Flg epitope is not restricted to different plant families; variations have also been found between species in the same family. A 15 aa peptide derived from *E. coli* Flg was shown only to be highly active in tomato (*Solanum lycopersicum* previously called *Lycopersicon esculentum*), but not in tobacco. Furthermore, the tomato Flg receptor, SlFLS2, an ortholog of the *Arabidopsis* FLS2 receptor, has now been identified and used in expression studies with *Nicotiana benthamiana*, where *N. benthamiana* expressing SlFLS2 gained the Flg perception system specific for tomato (Robatzek et al., 2007). In addition to this, studies focusing on host recognition of *Xanthomonas campestris* pv. *campestris* (*Xcc*) Flg have revealed within species and within pathovar variations for defense eliciting activity of Flgs among *Xcc* strains (Sun et al., 2006). Confirmation of FLS2 as a surface receptor came with studies using transgenic *Arabidopsis* Ws-0, expressing FLS2 fused to the green fluorescent protein (GFP), which revealed a cell membrane localization of FLS2. Additionally, FLS2 was found to undergo ligand-induced endocytosis; it is thought that this subcellular redistribution of FLS2, or any other surface receptor, from the plasma membrane to cytoplasmic vesicles may be a central point in signaling during immune responses (McCoy et al., 2004; Robatzek et al., 2006). Flg-induced activation of FLS2 in *Arabidopsis*, involves a complex formation with the Brassinosteroid-Insensitive 1 (BRI1)-associated receptor kinase 1 (BAK1) (Chinchilla et al., 2007). BAK1 has also been reported to be involved in BRI1 endocytosis (Russinova et al., 2004). Furthermore, BAK1 is required for the immune responses triggered by multiple MAMPs other than Flg, including the bacterial elongation factor EF-Tu (see below) (Roux et al., 2011). The activities of MAP kinases (MAPK) were delayed and reduced or even absent in response to flg22 or elf18, a fully active EF-Tu derivative, in *bak1* mutants, compared to wild type plants. This indicates that BAK1 acts as a positive regulator of MAMP signaling in *Arabidopsis*. In addition, it was revealed that FLS2, after flg22 stimulation, interacts with BAK1 in a ligand-dependent manner (Chinchilla et al., 2007). This interaction allows phosphorylation and activation of the receptor complex (Schulze et al., 2010). Downstream of the FLS2-BAK1 receptor complex is a cytoplasmic receptor kinase *Botrytis*-induced kinase 1 (BIK1), which constitutively associate with FLS2. After FLS2-BAK1 dimerization, BIK1 dissociate from FLS2, possibly allowing BIK1 to phosphorylate downstream components, and thus linking the MAMP receptor complex to downstream intracellular signaling leading to MTI (Lu et al., 2010). However, the substrates of FLS2 and BAK1 kinases have yet to be identified, and how the MAMP signal is transmitted from the BAK1-associated receptor complexes at the plasma membrane to intracellular events is largely unknown.

### Elongation factor Tu (EF-Tu)

In protein biosynthesis, the ribosomes translate the sequence of nucleotides in mRNA into the sequence of aa's in a protein. During the phase of elongation the ribosome is associated with elongation factors. One such elongation factor is EF-Tu, the most abundant protein in the bacterial cell (Jeppesen et al., 2005). The elicitor activity is attributed to a highly conserved part of the N-terminus of EF-Tu, either a 26 or 18 aa peptide named elf26 or elf18. The perception of EF-Tu by the EF-Tu Receptor (EFR) is independent of Flg perception, as EF-Tu is active in plants carrying mutations in FLS2 (Kunze et al., 2004). Although many of the signaling components downstream of EFR and FLS2 are shared between them (see above). EF-Tu recognition has only been found to elicit innate immunity in members of the family *Brassicaceae* (Zipfel et al., 2006). Studies using crosslinking assays in *Arabidopsis* cells, confirmed that elf18 and flg22 bind to different high-affinity binding receptors. Nevertheless, elf18 and flg22 were found by microarray analysis to induce the same pool of genes, and also a common set of responses in *Arabidopsis*. In addition to this, a combined treatment with both MAMPs, elf26/18 and flg22, was shown to induce the same kinases without an additive effect (Zipfel et al., 2006). An EF-Tu insensitive *efr-1* mutant did not respond with an oxidative burst, increased ethylene biosynthesis or induced resistance to *Pseudomonas syringae* pv. *tomato* (*Pst*) DC3000 in response to EF-Tu-derived elicitors, whereas *Arabidopsis* Col-0 and the *fls2* mutant did respond to EF-Tu elicitors. Heterologous expression studies of EFR in *N. benthamiana*, a plant lacking a perception system for EF-Tu, resulted in *N. benthamiana* with a perception system for EF-Tu, confirming the role for EFR as a functional receptor for EF-Tu (Zipfel et al., 2006). In addition to this, *efr* mutants were found to be more susceptible to *Agrobacterium tumefaciens (At*)-mediated transformation than wild type plants, indicating that EF-Tu recognition and the subsequent defense responses reduce *Agrobacterium*-mediated plant transformation (Zipfel et al., 2006).

Similar to FLS2, EFR belongs to the RLK family and has an ECD with 24 LRRs, a single TM domain and an intracellular serine/threonine kinase domain (Zipfel et al., 2006). Both FLS2 and EFR are members of the subfamily LRR-XII of RLKs (Shiu and Bleecker, 2003). Besides FLS2 and EFR from *Arabidopsis*, the rice pathogen recognition receptor, XA21 (see below), which confers resistance to *Xoo* strains is also a member of the LRR-XII subfamily (Song et al., 1995; Shiu et al., 2004; Lee et al., 2006). In contrast to FLS2, but like XA21, N-glycosylation is required for EFR functionality. Mutation of a single predicted glycosylation site compromised elf18 binding despite correct localization of the mutated protein to the plasma membrane (Häweker et al., 2010).

### Peptidoglycan (PGN)

PGN, a molecule never found in eukaryotes, is an essential and unique membrane envelope component of all bacteria, making it an excellent target for the eukaryotic innate immune system [reviewed by McDonald et al. (2005); Dziarski and Gupta (2006)]. PGN, which provides rigidity and structure to the cell envelope of both Gram-positive and Gram-negative bacteria, is a complex molecule consisting of numerous glycan chains that are cross-linked by oligo-peptides. These glycan chains are composed of altering N-acetylglucosamine (GlcNAc) and N-acetylmuramic acid (MurNAc), with short peptides attached by an amide linkage to the lactyl group of MurNAc. Several types of PGN, classified by the nature of the third residue of the stem peptide are commonly found. Typically, this is *m*-diaminopimelic acid (*m*DAP) PGN in Gram-negative bacteria and in some Gram-positive bacilli (genus *Bacillus* and *Clostridium*), whereas most other Gram-positive bacteria have L-lysine (LYS) PGN. In a recent study in tomato Nguyen et al. (2010) showed that pre-inoculation into tomato with *Staphylococcus aureus* PGN reduced the growth of a subsequent bacterial infection in PGN-treated tissue. This priming of defense with a MAMP is similar to that previously described for LPS (Newman et al., 2002). Early experiments with plant cells showed that the Gram-positive human pathogen *S. aureus* PGN was active as an elicitor in inducing extracellular alkalization of cultured tobacco cells, while no response was observed in cultured tomato cells, suggesting a different perception system for PGN within the *Solanaceae* (Felix and Boller, 2003a). PGN from both Gram-positive and Gram-negative bacteria was later found to act as elicitors of plant innate immunity in *Arabidopsis* (Gust et al., 2007; Erbs et al., 2008a). Gust et al. (2007) showed that it was the sugar backbone of the Gram-positive *S. aureus* PGN that was responsible for triggering immune responses and not the breakdown product of PGN, the muramyl dipeptide (MDP) or the muropeptide dimer, which is known to be the minimal chemical structure required for triggering the innate immune system in vertebrates and insects [reviewed by Traub et al. (2006)].

Erbs et al. (2008a), on the contrary, using PGN from two Gram-negative bacterial plant pathogens, *Xcc* and *At* found that both *Xcc* and *At* PGN and its constituents functioned as MAMPs in *Arabidopsis* and induced immune responses such as generation of ROS, extracellular pH increase, *PR1* gene expression, and callose deposition. Furthermore, they showed that the muropeptides were significantly more effective at inducing defense responses than the intact PGN molecule. These observations could be indicative of different perception systems for PGN from Gram-positive and Gram-negative bacteria or differences in structures, and therefore recognition sites, of the muropetides of human vs. plant pathogens. So far, the full structure of PGN from a Gram-positive plant pathogen has not been elucidated. In a study from 2009, Gimenez-Ibanez et al. showed that PGN from the bacterial pathogen *Pst* DC3000 induced the generation of ROS in *Arabidopsis cerk1* (chitin elicitor receptor kinase1) mutant plants, which indicated that *Pst* DC3000 PGN perception is independent of CERK1. In contrast, Willmann et al. (2011) reported that two of three *Arabidopsis* chitin oligosaccharide elicitor-binding proteins (AtCEBiP), LYM1, and LYM3, are involved in the perception/signaling of PGN (from various sources) together with AtCERK1, indicating the presence of a two-component receptor system similar to the rice chitin receptor OsCEBiP and OsCERK1 (Shimizu et al., 2010) (also see text below). All three proteins are required for PGN perception *in vivo* and for resistance to bacterial pathogens. Willmann et al.'s findings also showed that AtCERK1 is involved in the perception of at least two MAMP molecules, chitin and PGN in *Arabidopsis*. Interestingly, Shinya et al. (2012) showed that AtCERK1 serves both for chitin and PGN signaling, but AtCERK1 seems to contribute differently to the signaling. In the case of PGN signaling, the binding proteins LYM1 and LYM3 not only bind the ligand, but also contribute to the activation of AtCERK1 and downstream signaling, similarly to the function of OsCEBiP in rice. On the other hand, in the case of chitin signaling, AtCERK1 seems to function for both ligand perception and signaling (also see text below). Structurally, CEBiP is a receptor protein that contains extracellular LysM domains that are ~40 aa in length lacking a recognizable intracellular signaling domain. The LysM domains are considered to generally mediate binding to GlcNAc-containing glycans, like chitin and the backbone of PGN [reviewed by Gust et al. (2012)]. Also in monocots two LysM-containing PRRs have been shown to recognize PGN. Liu et al. (2012) reported two homologous rice lysine-motif containing proteins, LYP4 and LYP6. Both proteins bound PGN and chitin, but not LPS *in vitro*. Silencing either of the two proteins impaired the PGN or chitin-induced defense responses, and compromised the resistance against *Xoo* or the fungal pathogen *Magnaporthe oryzae*. These results suggest that PGN and chitin have overlapping perception components in rice.

In mammals, the recognition of PGN is complex, *e*.*g*., different receptors are found for PGN (extracellularly) and muropeptides (intracellularly). The cytosolic protein Nod2 can recognize MDP, from both Gram-positive and Gram-negative bacteria, and a lysine-containing muramyl tripeptide, but not a DAP-containing muramyl tripeptide (Girardin et al., 2003). In contrast, Nod1 only detects DAP-containing muropeptides. For instance, the human Nod1 recognizes the DAP-containing GlcNAc and MurNAc tripeptide (Chamaillard et al., 2003). The structure of the mammalian NOD proteins is similar to that of the plant R proteins, which are intracellular receptor proteins of the NB-LRR type (Inohara et al., 2005). In plants these proteins are involved in the recognition of specialized pathogen effectors leading to ETI (see above). However, in animals they seem to be involved in MAMP recognition rather than recognition of pathogen effectors. The future will show if a system similar to the Nod system, detecting intracellular microbial molecules, could be a possibility in plants.

### Lipopolysaccharides (LPS)

LPS, the major component of the outer membrane of Gram-negative bacteria, have been shown to have multiple roles in plant microbe interactions; it is thought to contribute to the restrictive Gram-negative membrane permeability, allowing bacterial growth in unfavorable environments. LPS and its derivatives act as MAMPs and induce innate immune responses in plants (Newman et al., 1995; Dow et al., 2000; Bedini et al., 2005; Silipo et al., 2005). Earlier studies in plants have shown that LPS can prevent the HR induced by bacteria. Pre-treatment of *Arabidopsis* leaves with LPS and its derivatives was found to prevent the HR caused by strains of *Pst* carrying the *avrRpm1* or the *avrRps4* genes, a phenomenon referred to as localized induced response (LIR; Newman et al., 2002; Silipo et al., 2005). The mechanisms behind HR prevention are still unknown, but the effects of LPS pre-treatment are considered to be associated with enhanced resistance of the plant tissue to pathogenic bacteria, which is thought to occur through an LPS-dependent potentiation of plant defense responses (Newman et al., 2002). LPS consists of a lipid, a core oligosaccharide, and an O-polysaccharide part. The lipid, referred to as lipid A, is embedded in the outer part of the phospholipid bilayer in the bacterial membrane. Lipid A and the core oligosaccharide are linked, usually by the sugar 3-deoxy-d-manno-2-octulosonate (KDO). The core oligosaccharide consists of a short series of sugars and ends in the O-antigen, which is composed of repeating oligosaccharide units (Raetz and Whitfield, 2002). The O-antigen of the LPS from many phytopathogenic bacteria has shown to consist of oligorhamnans (Bedini et al., 2002).

In order to know more about the structures within LPS that trigger immune responses in plants, synthetic O-antigen polysaccharides, oligorhamnans of increasing chain lengths, were tested in *Arabidopsis*. Tri-, hexa-, and nonasaccharides were synthesized and found to suppress the HR, as well as act as MAMPs and elicit the induction of the PR genes *PR1*and *PR2* in *Arabidopsis*. The efficiency of HR suppression and *PR* gene induction improved with increasing chain lengths of sugars in the synthetic O-antigen. In addition, a coiled structure was observed with the increasing chain length, indicating a role for this structure as a MAMP and by correlation a role for the O-antigen from phytopathogenic bacteria in plant innate immunity (Bedini et al., 2005). Studies in mammalian cells have shown that LPS is recognized through their lipid A moiety and this recognition was shown to govern the interactions with the innate immune system (Loppnow et al., 1989). In addition to this, the molecular shape of lipid A was found to directly correlate with its activity as a conical shape of lipid A was associated with endotoxicity and a cylindrical shape with antagonistic activity. A net negative charge of lipid A was found to influence its molecular conformation, and with that, its biological activity (Schromm et al., 1998, 2000). To study if the innate immune system from the mammalian system has parallels in the plant system, the role and mechanisms of action of LPS and its derivatives, the core oligosaccharide and the lipid A moiety, in plant-bacteria interactions were investigated in *Arabidopsis*. Initially, the complete structure of purified *Xcc* lipo-oligosaccharides (LOS), LPS without the O-chain, was determined. *Xcc* LOS was found to be a unique molecule with a high negative charge density and a phosphoramide group never found in such molecules before (Silipo et al., 2005). *Xcc* LOS and derivatives have been shown to elicit induction of the *PR* genes in *Arabidopsis.* LOS was found to induce the defense-related *PR1* and *PR2* genes in two temporal phases: the core oligosaccharide induced only the early phase and the lipid A moiety only the later phase, which suggests that both the core oligosaccharide and the lipid A are recognized by plant cells, e.g., both act as elicitors. These findings support the role of *Xcc* lipid A and the *Xcc* core oligosaccharide as MAMPs of innate immunity in plants. Silipo et al. (2005) speculated that the different LPS fragments are recognized by different plant receptors. This elicitor activity of *Xcc* lipid A correlates with earlier studies by Zeidler et al. (2004), who showed that lipid A preparations from various bacteria induced a rapid burst of NO production that was associated with the induction of defense-related genes in *Arabidopsis*. In a recent study by Madala et al. (2011) where the structure of *Burkholderia cepacia* strain ASP B 2D lipid A was determined, the role of lipid A as a MAMP in *Arabidopsis* was confirmed, and it was found to induce transcriptional changes associated with plant defense responses. Contrary to this, studies in tobacco cells, have shown that neither the lipid A nor the O-chain of the *Xcc* LPS molecule could induce the oxidative burst alone, but rather it was the inner core part of the LPS molecule that was responsible (Braun et al., 2005). The conflict in results could reflect the different defense responses measured after treatment with LPS and its derivatives in different plants.

In correlation to studies in the mammalian system, where it is well-established that the phosphorylation pattern of lipid A affects its biological activity [reviewed by Gutsmann et al. (2007)], it was tested whether de-phosphorylated *Xcc* LOS could be recognized in plants. After de-phosphorylation of *Xcc* LOS the molecule maintained only the negative charge of the KDO residue, and rendered the molecule unable to induce LIR, suggesting that the charged groups present in LOS play a key role in inducing defense responses in *Arabidopsis* (Silipo et al., 2005). Furthermore, from these experiments it could be concluded that the electrostatic interactions involving the phosphate groups seem to have a crucial function in binding not only lipid A, but also the core oligosaccharide, to putative receptors in plants (Silipo et al., 2005). LPS has been found, not only to induce defense responses, but also to prime expression of plant defense responses upon subsequent bacterial inoculation, e.g., promote an early triggering of the synthesis of the antimicrobial compounds feruloyl tyramine (FT) and p-coumaroyl tyramine (CT) (Newman et al., 2002, 2007; Prime-A-Plant Group, 2006). The O-antigen part of the LPS molecule is thought to be responsible for induced systemic resistance (ISR) in *Arabidopsis*. Early studies showed that LPS from the rhizobacterium *Pseudomonas fluorescens*, as well as the live bacteria, induced ISR in carnation and radish, whereas mutant bacteria, lacking the O-antigen side chain could not induce ISR (Leeman et al., 1995; van Loon et al., 1998). In contrast to the rhizobacteria-mediated ISR, systemic activation of defense-related responses in plants upon local necrotizing pathogen infection is referred to as systemic acquired resistance (SAR). SAR is accompanied by a systemic increase in salicylic acid (SA), and SA is required for SAR signaling (Ryals et al., 1996; Schneider et al., 1996). However, recent studies suggest that recognition of the MAMPs, LPS, or Flg, and not necrotic lesion formations contribute to the bacterial induction of SAR in *Arabidopsis*. Treatment of *Arabidopsis* with *P. aeruginosa* LPS, Flg or non-host bacteria were shown to be associated with accumulation of SA, expression of the *PR* genes and expression of the SAR marker gene *Flavin-dependent monooxygenase 1* in treated as well as in distant leaves (Mishina and Zeier, 2006, 2007). The signaling cascades underlying SAR and NO production after perception of LPS by plant cells have not yet been resolved. Sun et al. (2012) investigated the biosynthetic origin of NO and the role of Non-expressor of Pathogenesis-Related Genes (NPR1) to gain insight into the mechanism involved in LPS-induced resistance of *Arabidopsis*. NPR1 is a key regulator of SAR, and is essential for SA signal transduction (Rockel et al., 2002). Analysis of inhibitors and mutants showed that LPS-induced NO synthesis was mainly mediated by an arginine-utilizing source of NO generation. LPS (Sigma)-activated defense responses, including callose deposition and defense-related gene expression, were found to be regulated through an NPR1-dependent pathway. In contrast, *Xcc* LPS can induce defense responses in pepper without triggering the oxidative burst or SA synthesis (Newman et al., 2002).

The activity of LPS in plants has mostly been described in dicots, but studies in rice cells have revealed that LPS, from various pathogenic and non-pathogenic bacteria, induce a generation of ROS and defense-related gene expression in monocots, indicating that the machinery recognizing LPS is evolutionary conserved in monocots and dicots (Desaki et al., 2006, 2012). Furthermore, the two MAMPs, LPS and chitin oligosaccharide, induced a close correlation of genes in rice cells, indicating a convergence in signaling cascades downstream of recognition. In addition, the effect of LPS from various bacteria was shown to be associated with a programmed cell death (PCD) in rice cells. In contrast, LPS has never been shown to elicit PCD in dicots (Desaki et al., 2006). The mechanism by which LPS is perceived by plants is still not understood. Recent studies with fluorescein-labeled *Xcc* LPS in cultured *N. tabacum* cells revealed that LPS was rapidly bound to the cell wall and then internalized into the cell, and eventually, LPS was found exclusively inside the vacuole. These findings suggest endocytosis, comparable to the mammalian system, of *Xcc* LPS in tobacco cells (Gross et al., 2005). However, no PRRs for LPS and its derivatives have been characterized in plants. In the mammalian immune system, LPS form complexes with LPS-binding proteins (LBP), and this LPS-LBP complex is recognized by the membrane-bound CD14 receptor, glycosylphosphatidyl inositol (GPI)-anchored glycoprotein (Wright et al., 1990), which again is thought to associate with TLR4-MD2 to participate in LPS-induced signaling (Jiang et al., 2000; Miyake, 2004). The concentrations of LPS required to elicit most of the effects described above are in the 5–100 μg per ml range, suggesting that plants do not have the sensitivity to LPS shown by mammalian cells, which can respond at concentrations in the pg to ng per ml range. These considerations have led to suggestions that plants possess only low affinity systems to detect LPS (Zeidler et al., 2004), although plants can detect other bacterial MAMPs such as the peptides derived from Flg and EF-Tu elongation factor at sub nM levels. One complicating factor is the aggregation of LPS molecules within the purified preparations, which may affect the ability of LPS to cross the matrix of the plant cell wall to reach presumed membrane-associated receptors (Aslam et al., 2009).

Many groups have attempted to identify plant components involved in LPS recognition and perception often with conflicting results. Livaja et al. (2008) found that in *Arabidopsis* cells, *B. cepacia* LPS induced a leucine-rich repeat RLK At5g45840 by nearly 17-fold after 30 min. Furthermore, in a proteomic analysis of the changes following perception of LPS from an endophytic strain of *B. cepacia* in *N. tabacum* BY-2 cells, 88 LPS induced/regulated proteins, and phosphoproteins were identified, many of which were found to be involved in metabolism and energy-related processes. Moreover, proteins were found that are known to be involved in protein synthesis, protein folding, vesicle trafficking, and secretion (Gerber et al., 2006, 2008). In a transcription profiling of *A. thaliana* cells treated with LPS from *B. cepacia*, Livaja et al. (2008) surprisingly did not find any genes involved in callose synthesis. Furthermore, genes involved in ROS production were found to be upregulated at a very low level by *B. cepacia* LPS. In addition, Livaja et al. (2008) found that *B. cepacia* LPS only induced the *PR* genes *PR3* and *PR4*, whereas studies in *B. cepacia* LPS treated *Arabidopsis* leaves revealed induction of several *PR* genes (Zeidler et al., 2004). Other LPS preparations, from *P. aeruginosa* and *Escherichia coli*, respectively, induce *PR1* and *PR5* in *Arabidopsis* leaves (Mishina and Zeier, 2007). The variation in results both reflects the different plant systems (*Arabidopsis* cell cultures contra the whole plant) and the origin of the LPS. All the above very specific effects show the ability of particular plants to recognize structural features within LPS that are not necessarily widely conserved.

Recognition of LPS/LOS in mammals is rather complex; how complex this recognition is in plants is still not known, and the mechanism of this recognition and consequent transduction steps in plants remains obscure. Gross et al. (2005) showed that, in tobacco cells, *Xcc* LPS was internalized 2 h after its introduction to a cell suspension, where it co-localized with Ara6, a plant homolog of Rab5 which is known to regulate early endosomal functions in mammals. It was speculated that this endocytosis in tobacco cells was, in correlation with the mammalian system, part of a down regulation of defense responses. In a recent study by Zeidler et al. (2010) localization and mobilization of fluorescein-labeled *Salmonella minnesota* LPS was studied in *Arabidopsis*. Leaves were pressure infiltrated with fluorescein-labeled *S. minnesota* LPS and the mobility of LPS was studied over time by fluorescence microscopy. After 1 h a fluorescent signal was observed in the intercellular space of the infiltrated leaf. The labeled LPS were visible in the midrib of the leaves after 4 h, whereas this fluorescence had spread to the smaller leaf veins near the midrib after 6 h. After 24 h it was detectable in the lateral veins. Moreover, cross-sections of the midrib 3 h after supplementation with fluorescein-labeled LPS revealed a fluorescent signal in the xylem. Using capillary zone electrophoresis a distribution of fluorescein-labeled *S. minnesota* LPS was found in the treated as well as in systemic leaves of the plant (Zeidler et al., 2010). In contrast to the results reported by Gross et al. (2005), no intracellular accumulation of the labeled LPS was observed in *Arabidopsis*. This disparity in outcomes might again be a reflection of the use of different plants, the difference in the age of the plants used (plant cell cultures vs. seedlings vs. fully developed plants) and the different defense responses measured after treatment with LPS and its derivatives.

Alterations in lipid A or other structures within LPS are known to occur during symbiotic interactions with plants (Kannenberg and Carlson, 2001) and in response to compounds in plant root exudates (Fischer et al., 2003) and may occur during plant pathogenesis. These alterations may serve both to increase the resistance of the bacteria against host defenses and to attenuate the activity of lipid A or LPS in triggering those defenses. Characterization of the structure and function of LOS from a non-pathogenic *Xcc* mutant strain 8530, which carries a Tn5 insertion in a gene of unknown function (Dow et al., 1995), revealed that this mutant had a truncated core region. The fact that *Xcc* strain 8530 was defective in core completion led to significant modifications in the acylation and phosphorylation patterns of its lipid A, and these changes had influence on its ability to trigger innate immune responses in *Arabidopsis* (Silipo et al., 2008). The core sugars provide protection against antimicrobial compounds and attenuate the endotoxic properties of lipid A, similar to lipid A modifications seen in mammalian pathogens (Raetz et al., 2007). These findings indicate that *Xcc* has the capacity to modify the structure of lipid A and thus reduce its activity as a MAMP in plants (Silipo et al., 2008). The acyl chains of lipid A can vary, as can the number and length of them depending on growth conditions and bacterial species. Studies in mammalian cells have shown that LPS from *Shigella flexneri* elicit a weaker TLR4-mediated response than *E. coli* LPS due to differences in the acylation status of their lipid A moieties (Rallabhandi et al., 2008).

Lipid A from *Halomonas magadiensis*, an extremophilic and alkaliphilic Gram-negative bacteria, isolated from a soda lake in an East African Rift Valley has been found to act as an LPS antagonist in human cells (Silipo et al., 2004). *H. magadiensis* lipid A, characterized by an unusual and very low degree of acylation, was verified to inhibit *E. coli* lipid A-induced immune responses in human cells (Ialenti et al., 2006). *E. coli* lipid A, which is an effective agonistic structure of immune responses in mammalian cells, is composed of a bis-phosphorylated hexa-acylated disaccharide backbone with an asymmetric distribution of the acyl residues. Studies have revealed that structural differences on the lipid A skeleton, for example, acylation can affect its agonist/antagonist activity (Munford and Varley, 2006). In consonant with the ability in blocking enteric LPS-induced human monocyte activation, our laboratory found that *H. magadiensis* lipid A was able to antagonize the action of *E. coli* lipid A when inducing *PR1* gene expression in *Arabidopsis*. Even though the mode of perception of LPS in plants is far less-understood than in mammals and insects, these results indicate that *Arabidopsis* is sensitive to the same structures of lipid A that determine biological activity in humans (Erbs et al., 2008b).

Thus far, LPS preparations used for the analysis of plant responses and for structural studies have been derived from bacteria grown in culture. We know nothing about the alterations in LPS that might occur when bacteria are within plants, although this may be highly relevant for recognition and signaling. Changes could occur in both the size distribution of LPS (alteration in the ratio of LOS to LPS) and/or in decoration of LPS with saccharide, fatty acid, phosphate, or other constituents. Increases in the sensitivity of mass spectrometric methodologies may allow development of micro-methods to analyse such changes in bacteria isolated from plants. Transcriptome or proteome profiling of bacteria isolated from plants may also give clues as to possible LPS modifications.

Intriguingly, although lipid A-like molecules have not been reported in plants, many plants, including *Arabidopsis*, encode full-length nuclear orthologs of six of the nine enzymes of the *E. coli* biosynthetic genes for lipid A. *Arabidopsis* mutants generated by knock-out of these genes are viable under laboratory conditions. However, they accumulate (wild type) or lose (mutant) the expected lipid A precursors (Li et al., 2011). The lipid A biosynthetic genes of higher plants may have been acquired from Gram-negative bacteria with the endosymbiosis of mitochondria. Plant lipid A may therefore play a structural role in mitochondrial or perhaps chloroplast membranes. Alternatively, lipid A-like molecules in *Arabidopsis* may be involved in signal transduction of plant defense responses. Although the mechanisms by which plants detect LPS remain unknown, lipid A-like molecules in plants might serve as signals to regulate cellular responses during plant pathogen invasion.

## Activator of XA21-mediated immunity (Ax21)

Even though the rice receptor XA21 has been known for a long time, the corresponding ligand Ax21 (previously known as avrXa21) was identified only recently (Lee et al., 2009). The conservation of Ax21 in all sequenced *Xanthomonas* spp., *Xylella fastidiosa*, and the human pathogen *Stenotrophomonas maltophila* suggests that it plays a key role in a biological function. *Ax21* encodes a 194 aa protein (Bogdanove et al., 2011). The minimal recognized epitope mimicking Ax21 activity is a 17 aa sulfated peptide, called axY^s^22, which has been shown to be 100% identical among six different *Xanthomonas* spp. (Lee et al., 2009). XA21, is, together with FLS2 and EFR, among the best studied PRRs, they all belong to subfamily LRR XII of the non-RD class of receptor kinases (Shiu and Bleecker, 2003; Shiu et al., 2004; Dardick and Ronald, 2006). *Xa21* was originally identified as a dominant-resistant locus conferring resistance to multiple *Xoo* races in the wild rice species *O. longistaminata* (Khush et al., 1990). *Xa21* maps to chromosome 11, and already upon its discovery it was speculated to encode a gene product recognizing a determinant present in all *Xoo* races (Ronald et al., 1992). Later, the resistance of locus *Xa21* was linked to a single gene, also named *Xa21*, encoding a receptor kinase-like protein with predicted LRR, TM, juxtamembrane (JM), and intracellular kinase domains and that this single gene was sufficient to confer resistance to a number of *Xoo* isolates (Song et al., 1995; Wang et al., 1996). *Xa21* is a member of a gene family with at least seven members in rice. The closest relative to *Xa21* is *Xa21D* and the spectrum of resistance is identical between the two genes, but the level of resistance differs as *XA21D* only confers partial resistance. The LRR domains of Xa21 and Xa21D are more than 99% identical, but Xa21D lacks the TM and kinase domains and it may therefore have an extracellular function, but the mode of action is unknown (Song et al., 1997; Wang et al., 1998).

Several proteins have been shown to interact with XA21. The ATPase XB24 (XA21-binding protein 24) promotes autophosphorylation of XA21, which is thereby kept in an inactive state. When Ax21 binds to XA21, the XB24/XA21 protein complex probably dissociates, and XA21 is activated (Chen et al., 2010). After activation, the phosphatase XB15 (XA21-binding protein 15) dephosphorylates XA21 in order to deactivate it again (Park et al., 2008). A recent study has shown that upon Ax21 recognitions by XA21, the intercellular kinase domain is released and translocated to the cell nucleus, a translocation that is necessary for the XA21-mediated immune response (Park and Ronald, 2012). The rice transcription factor OsWKKY62 (also called XB10), which has previously been shown to be a negative regulator of XA21 activity, is needed for this translocation (Peng et al., 2008; Park and Ronald, 2012). The E3 ubiquitin kinase XB3 (XA21-binding protein 3) is important for XA21-mediated resistance, as rice lines silenced in XB3 both has a decreased level of XA21 and display reduced resistance to *Xoo* (Wang et al., 2006). Less thoroughly studied are the genes *Rox1, 2*, and *3* (Regulator of XA21-mediated immunity 1, 2, and 3 encoding a thiamine phosphokinase, a NOL1/NOL2/sun gene family member and a nuclear migration protein, respectively), which have also been shown to affect *Xoo* resistance in XA21-containing rice plants (Lee et al., 2011).

Interestingly, the *Arabidopsis* FLS2 and EFR receptors bind the artificial Ax21 derived peptide axY^s^22-A1, this binding trigger responses similar to the ones triggered by Flg. axY^s^22-A1 is identical to axY^s^22, except that the first aa in the peptide has been changed from Ala to Glu (Danna et al., 2011). It was previously thought that FLS2 was specific to Flg. Even though the authors analyzed their axY^s^22-A1 peptide stocks for the presence of flg22 by mass spectrometry, a question was later raised whether the observations were caused by flg22 contamination (Danna et al., 2011; Mueller et al., 2012). Mueller et al. (2012) had observed incidences of commercially produced peptides contaminated with flg22 and even minute amounts (in the range of 1 ppm) will activate FLS2 responses. Furthermore, *Arabidopsis* cell cultures did not respond to treatment with axY^s^22 in their laboratory, therefore they concluded that the FLS2 binding observed by Danna et al. (2011) could be caused by contamination (Mueller et al., 2012). These concerns were dismissed by Danna et al. (2012), who believe that the difference in peptides used (axY^s^22-A1 vs. axY^s^22) and the differences in their experimental set-ups, could explain different results.

Ax21 is secreted from the bacterial cell through the TOSS—a fact that has been known longer than the identity of Ax21 (da Silva et al., 2004). TOSS is a relatively simple system consisting of only three protein subunits: a membrane fusion protein (MFP), an adenosine triphosphate-binding cassette (ABC) transporter, and an outer membrane protein (OMP). Three *Xoo* genes with homology to the TOSS components have been shown to be required for Ax21 activity (the so called *rax* genes). The genes *raxA, raxB*, and *raxC* are identified as coding for a MFP, an ABC transporter, and an OMP, respectively (da Silva et al., 2004). *raxA* and *raxB* are arranged in a putative operon (called *raxSTAB*) together with the gene *raxST*, which is not a part of the TOSS (da Silva et al., 2004). Instead *raxST* is a sulfotransferase (Shuguo et al., 2012), which catalyze the transfer of sulfate from PAPS (3′-phosphoadenosine 5′-phosphosulfate) to a tyrosine residue. Sulfation of secreted peptides is often important for their biological function. This is also the case for the Ax21-derived peptide axY^s^22, as a non-sulfated version of the peptide, axY22 is not recognized by XA21 (Lee et al., 2009). Based on genetic similarity and complementation studies, the two genes *raxP* and *raxQ* have been suggested to be responsible for the PAPS synthesis as they encode proteins with ATP sulfohydrolase and APS kinase activities (Shen et al., 2002). Downstream of *raxSTAB* another putative operon, comprised of the two genes *raxR* and *raxH*, has been found. *Xoo* strains mutated in these two genes do not express full Ax21 activity. These two genes are probably encoding proteins involved in a bacterial two component regulatory system (a response regulator and a histidine protein kinase), and they could be involved in the regulation of a number of genes (Burdman et al., 2004). The two component system composed of RaxR and RaxH have also been found to regulate the expression of another two component system composed of PhoP and PhoQ. The PhoPQ system also seems to control the TTSS, important for delivering bacterial effector molecules to the host cell, through regulation of the *hrpG* gene (Lee et al., 2008).

As Ax21 was shown to be a secreted molecule (Lee et al., 2009) and the finding that the expression of *raxST, raxP, raxR*, and *raxC* are density-dependent it was suggested that Ax21 is a quorum sensing (QS) molecule (Lee et al., 2006). This was supported by a finding in *S. maltophila*, showing that mutants lacking Ax21 display reduced motility and biofilm formation. Also in this organism it appears that RaxH and RaxR are part of a two-component system (McCarthy et al., 2011). Han et al. (2011) published the evidence for this hypothesis, thereby making Ax21 the first QS factor also functioning as a MAMP. But unfortunately critical errors of a central *Xoo* strain used in the study has been found and the authors of the original paper are now in the process of repeating the experiments with a new validated strain in order to confirm the results (Comment on the PLoS homepage since January 2013). The outcome of these experiments should be followed with great interest. Knowledge of detection of small proteins, like QS in rice and other species, can be used to develop reagents to disrupt QS-mediated virulence activities.

## Fungal and oomycete MAMPs

### Chitin and β-glucan

Examples of MAMPs from fungi and oomycetes include the fungal chitin and β-glucan from *P. megasperma*. However, data describing how these MAMPs are recognized and how the following signal transduction is mediated has only in a few cases been accomplished.

In fungal cell walls branched β-glucan is cross-linked to chitin and in oomycetes to cellulose. In soybean the PRR recognizing *P. megasperma* β-glucan was identified as the β-glucan binding protein (GBP) (Umemoto et al., 1997). This MAMP and its corresponding PRR has not been studied further. On the other hand chitin and its fragments chitin oligosaccharides have been shown to trigger defense responses in both monocots and dicots. Together with CEBiP, CERK1 recognizes fungal chitin (Kaku et al., 2006; Shimizu et al., 2010). In rice, the RLP CEBiP binds chitin oligosaccharides at the cell surface and interacts with the LysM-RLK OsCERK1 for signaling. In *Arabidopsis*, only the LysM-containing RLK CERK1 was found to be essential for chitin elicitor signaling (Miya et al., 2007). Three CEBiP-like proteins, LYM1-3, have been identified in *Arabidopsis*. Using heterologous expression of these three *Arabidopsis* CEBiP homologous in tobacco BY-2 cells Shinya et al. (2012) tested for their ability to bind chitin oligosaccharides and found that only LYM2, also referred to as AtCEBiP, showed high affinity binding to chitin oligosaccharides. Even though affinity labeling with biotinylated (GlcNAc)_8_ indicated that AtCEBiP represent a cell surface chitin-binding protein, knockout (KO) of AtCEBiP, LYM1, or LYM3, single or triple KO, together with AtCEBiP overexpression studies suggested that AtCEBiP does not contribute to chitin signaling in *Arabidopsis* (Shinya et al., 2012). These studies reveal that *Arabidopsis* and rice exploit different chitin receptor systems. Similar results were obtained by Wan et al. (2012) who showed that mutations in each of the three *Arabidopsis* CEBiP-like proteins 1, 2, or 3, or in a combination resulting in a triple mutant, had no effect on the plant response to chitin. *Arabidopsis* has five LysM RLKs1-5 (Lyk1, 2, 3, 4, and 5) one of them, Lyk1, is also known as CERK1. Wan et al. (2012) tested the *Arabidopsis lyk2, 3, 4*, and *5* KO mutants, respectively, to see if they were involved in chitin signaling. They found that the plant immune response to chitin was reduced only in the *lyk4* mutant suggesting Lyk4 to be involved in a chitin recognition receptor complex (Wan et al., 2012). Furthermore, the *lyk4* plants were more susceptible to the fungal pathogen *Alternaria brassicicola* and the bacterial pathogen *Pst* DC3000 than wild type plant (Wan et al., 2012). In addition to this it has been reported that two rice lysine-motif containing proteins, LYP4 and LYP6, could bind both PGN and chitin acting as dual functional PRRs in rice innate immunity (Liu et al., 2012). Their results further suggest that overlapping perception systems exist for bacterial PGN and fungal chitin in rice. In contrast, LYM1 and LYM3 the orthologs of LYP4 and LYP6 in *Arabidopsis* were only able to bind PGN and not chitin (Willmann et al., 2011). Further details on PGN recognition can be found in the text above.

### Ave1 peptide and ethylene-inducing xylanase (EIX)

In tomato a *Verticillium* resistance locus *Ve* was identified that mediates resistance against race 1 strains of *Verticillium dahlia* and *V. albo-atrium*, respectively (Kawchuk et al., 2001). The characterization of the *Ve* locus revealed two genes *Ve1* and *Ve2* that encode cell-surface receptors belonging to the LRR class of RLP. Only *Ve1* was found to confer resistance in tomato. Moreover, tomato plants silenced in *BAK1*, showed higher susceptibility to infection with *Verticillium* indicating that BAK1 is involved in Ve1-induced defense responses in tomato (Fradin et al., 2009). A putative ligand for the LRR-RLP Ve1 is the Ave1 (avirulence on Ve1 tomato) peptide. Ave1, a conserved peptide identified in several fungi and in the plant pathogenic bacteria *Xanthomonas axonopodis* pv. *citri*, has been found to have homology to plant natriuretic peptides (PNPs). PNPs are extracellular signaling molecules that have been shown to have a role in regulation of homeostasis under several stress conditions (Wang et al., 2011). Ave1 acts as an elicitor of disease resistance mediated by the LRR-RLP Ve1 in tomato (de Jonge et al., 2012), but a direct binding between Ave1 and Ve1 remains to be shown. Ve1 has been referred to as a PRR or an R protein accompanied by speculations that the Ave1 peptide could be an effector acting as a MAMP (Thomma et al., 2011). Future results will reveal, if it is possible to differentiate as strictly, as we do today, between MAMPs and effectors, as between PRRs and R proteins.

Two other PRRs in tomato, the LRR-RLPs SlEix1 and SlEix2, which have been shown to have homology to the tomato Ve and Cf PRRs, recognize the fungal ethylene-inducing xylanase (EIX) (Ron and Avni, 2004). EIX is a 22-kD fungal protein (β-1-4-endoxylanase) from *Trichoderma viride* that independent of its endoxylanase activity can act as an elicitor of defense responses in tomato and tobacco plants (Furman-Matarasso et al., 1999; Ron and Avni, 2004). The aa sequence of SlEix1 and SlEix2 are 81.4% identical, SlEix1 and SlEix2 both bind EIX, but their functions differ. The SlEix2 receptor has been shown to be internalized upon EIX application (Bar and Avni, 2009) and only SlEix2 transmits the signal mediated by EIX leading to plant immune responses (Ron and Avni, 2004). SlEix1, on the other hand, block the EIX signaling and the authors suggested that SlEix1 functions in inhibition of plant defense signaling and plant cell death in response to EIX (Bar et al., 2010). Using BAK1-silenced tobacco plants Bar et al. (2010) further showed that BAK1 was required for this inhibitory activity of SlEix1 on SlEix2 signaling and endocytosis.

### Damage-associated molecular patterns (DAMPs)

The plant defense system is not only recognizing microbial elicitors, some plant-derived molecules also induce plant defense responses. This sensing of infectious-self or modified-self is mediated by DAMPs (Seong and Matzinger, 2004; Boller and Felix, 2009), also referred to as microbe-induced molecular patterns (MIMPs, Mackey and McFall, 2006). Similarly the mammalian immune system detects “danger” through a series of DAMPs, now also in in this system named damage-associated. The mammalian DAMPs are derived from other tissues activating intracellular cascades that lead to an inflammatory response (Lotze et al., 2007).

In Plants the 18 aa tomato peptide systemin is an endogenous elicitor of plant defense (Pearce et al., 1991; McGurl et al., 1992). The systemin precursor prosystemin is a cytoplasmic protein and upon cell damage the released systemin acts as a DAMP on surrounding cells (Narváez-Vásquez and Ryan, 2004). Early reports showed that the RLK SR160 (the tomato ortholog of BRI1) was the receptor for systemin (Scheer and Ryan, 1999, 2002), but later reports found that null mutants were sensitive to systemin (Holton et al., 2007; Lanfermeijer et al., 2008). Also in *Arabidopsis* a system with a putative cytosolic peptide (Pep1) activates transcription of defense-related genes and induces alkalization in cell cultures. The 23 aa Pep1 and the seven homologous in *Arabidopsis* (Pep 1–7) are derived from the C-terminal part of their precursor proteins PROPEP1–7 (Huffaker et al., 2006). The Pep1 receptor, PEPR1, was found to be a LRR receptor belonging to the LRR XI subfamily (Yamaguchi et al., 2006). Based on sequence similarity a second receptor of Pep-peptides, PEPR2, has been identified. Transcription of both *PEPR1* and *2* is activated by wounding, Methyl-Jasmonate (MeJA), Pep-peptides, and specific MAMPs. A level of redundancy is found regarding ligand specificity of PEPR1 and 2 as they both bind Pep1 and 2, and in addition PEPR1 binds Pep3–6 (Yamaguchi et al., 2010).

Oligogalacturonides (OG) and cutin released from plant cell walls also function as DAMPs (Schweizer et al., 1996; Denoux et al., 2008). Using a domain swap approach Brutus et al. (2010) proved that WAK1 (Wall-Associated Kinase 1) function as an OG receptor whereas a receptor for cutin still remains to be found.

## Concluding remarks

Even though MAMPs are much conserved, they are under selective pressure in adapted pathogens to evade recognition. For example in the case of bacterial Flg, a potent inducer of MTI in most plants, mutations in key residues of the flg22 epitope that abolish recognition by the receptor FLS2 have been selected for in several plant pathogens and symbionts (Boller and Felix, 2009; Lopez-Gomez et al., 2012). Notably, this positive selection seems to be more rapid than previously thought, as modern natural isolates of *Pst* adapt to their tomato host through non-synonymous mutations in the Flg-encoding gene *fliC* (Cai et al., 2011). The best example so far of a glycosylated MAMP not being recognized in plants, is the LPS molecule from the nitrogen-fixing soil bacterium *Bradyrhizobium* sp. BTAi1, this LPS does not trigger the innate immune response in different plant families. *Aeschynomene indica* (the natural host of *Bradyrhizobium*), *Lotus japonicus*, and *Arabidopsis* were tested for perception of *Bradyrhizobium* LPS. Defense responses were not induced in any of the tested plants. The authors determined the structure of *Bradyrhizobium* LPS and found a unique LPS with an, in nature, unprecedented chemical structure of the monosaccharide forming the polymer, this “different” structure probably prevents recognition by the LPS receptor complex in plants (Silipo et al., 2011). MAMPs are necessary for microbial life and therefore under strong negative selection, but their immunogenic epitopes are under positive selection to evade host immune detection. These opposing evolutionary forces were recently used to identify novel candidate MAMPs from *Pseudomonas* and *Xanthomonas* species through an innovative bioinformatics approach. Identifying new MAMPs may prove to be a source of new antimicrobial agents (McCann et al., 2012).

Although plant receptors for bacterial PGN and the proteinaceous MAMPs Flg and EF-Tu elongation factor have been identified, those involved in perception of LPS remain obscure. In conclusion we expect that in the next few years we will see a substantial increase in our understanding of the processes of MAMPs perception and signal transduction in plants through the deployment of cross disciplinary approaches and ever expanding ranges of molecular experimental tools. Despite their critical role in immunity, we know remarkably little about the range and diversity of MAMPs. Most studies have focused on a limited number of MAMPs as described in this review. The identification of new MAMPs will give insight into the molecular and evolutionary mechanisms underlying host-pathogen interactions, and greater understanding of the mechanisms by which MAMPs elicits defense responses may have considerable impact on the improvement of plant health and disease resistance.

### Conflict of interest statement

The authors declare that the research was conducted in the absence of any commercial or financial relationships that could be construed as a potential conflict of interest.
